# Appendiceal diverticulum masquerading as acute appendicitis

**DOI:** 10.1093/jscr/rjac248

**Published:** 2022-05-31

**Authors:** A Patel, A Abbas, R Bhattacharyya, D Galaktionova

**Affiliations:** 1 School of Medicine, Rowan University School of Osteopathic Medicine, Stratford, NJ, USA; 2 St. Joseph’s University Medical Center, Department of Surgery, Paterson, NJ, USA

## Abstract

Appendiceal diverticula present as rare clinical finding and are most confused with acute appendicitis. A 65-year-old female was presented to our surgical service after 1 day of right lower quadrant abdominal pain and a computed tomography evaluation, read as a diagnosis of acute appendicitis. Due to the location and quality of pain and intraoperative findings acute appendicitis was our preliminary diagnosis. Follow up with histopathology confirmed acute inflammation of an appendiceal diverticulum. With discordance in original diagnosis from final pathological evaluation, we suggest appendiceal diverticula as an important differential to consider in patients of similar clinical presentation. Surgical treatment with appendectomy and final histological diagnosis are essential in the proper treatment of this rare clinical finding.

## INTRODUCTION

Appendiceal diverticula are confirmed as an incidental pathologic finding rarely reported amongst cases involving appendectomies with similar clinical presentation. It is frequently confused with acute appendicitis [1]. We present a rare case of a 65-year-old female treated for acute appendicitis who was instead found to have acute sequelae of appendiceal diverticulosis.

## CASE REPORT

**Figure 1:**
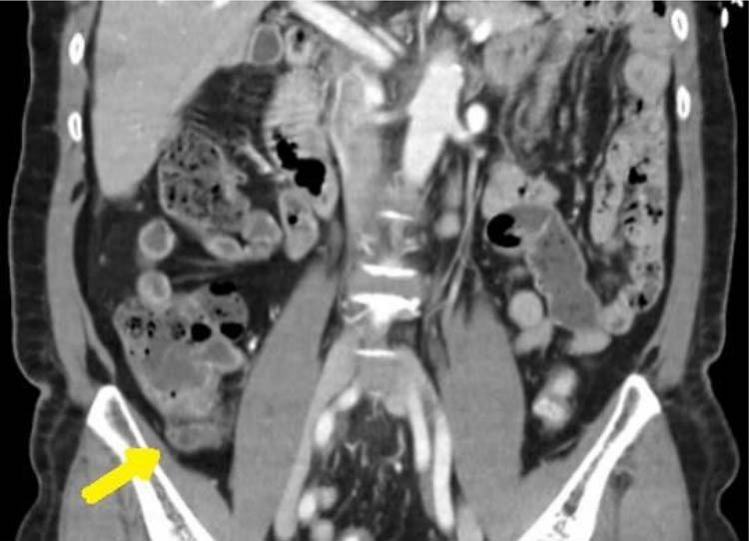
CT scan coronal view: dilated appendix with fat stranding.

A 65-year-old female with a past medical history of hyperlipidemia presented to the emergency department with 1 day of right lower quadrant (RLQ) abdominal pain. These symptoms were confirmed on physical examination as moderately tender to palpation. Clinical assessment was otherwise unremarkable. Yet, a computed tomography (CT) was obtained confirming high suspicion for acute appendicitis with an enlarged appendix of 9 mm and associated with wall enhancement and evidence of fat stranding (see Fig. 1).

**Figure 2:**
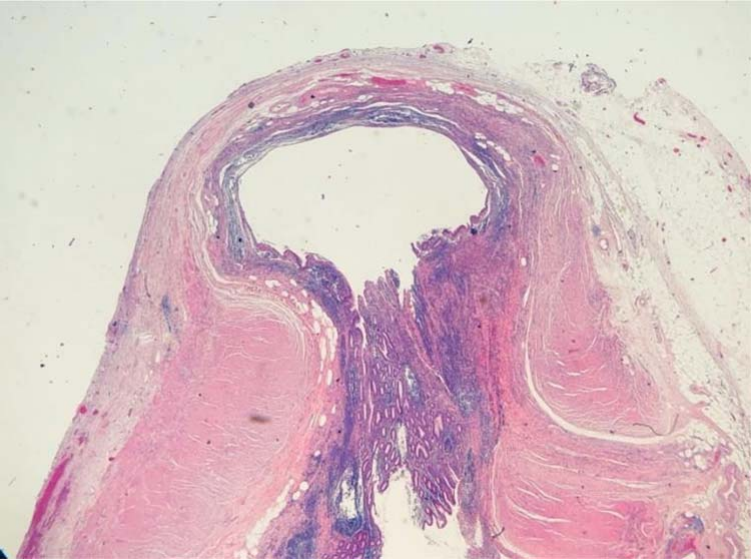
Pathology slide: diverticulosis of the appendix.

Given the clinical assessment and CT findings, the patient was taken to the operating room for a laparoscopic appendectomy, which was uncomplicated. During the procedure, copious adhesions were noted in the lower midline involving the omentum. The appendix was found to be edematous and hyperemic with no perforation. Pathological analysis demonstrated focal acute inflammation of the mucosa with a normal appendiceal wall (see Fig. 2). Patient was discharged home the same day following surgery with an unremarkable follow-up.

## DISCUSSION

We present a rare case of appendicitis with diverticulosis. The incidence in such cases is reported at a rate no greater than 1%. Diverticula of the appendix are commonly classified as congenital or acquired. The former being significantly less common. It is most found in men over the age of 30, which is not the typical demographic for acute appendicitis [2]. Very few are diagnosed prior to surgical pathology reporting [2–4]. Acquired diverticula are found more often than congenital diverticula, and are classified as false diverticula, as they arise from mucosal herniation through a weak point in the wall [5]. The etiology of diverticular disease of the appendix remains poorly understood, however it is hypothesized that uncoordinated muscular contractions due to luminal obstruction may increase intraluminal pressure, and ultimately produce mucosal herniation through the wall of the appendix [5].

Diverticula of the appendix are commonly identified incidentally post appendectomy for patients with a clinical presentation of acute appendicitis [1]. The Lipton classification suggests four subtypes of disease pertaining to such presentations of appendiceal diverticulitis [1]. Type I is acute diverticulitis of appendiceal diverticula; Type II is appendicitis combined with acute diverticulitis of appendiceal diverticula; Type III is appendicitis with an appendiceal diverticulum and Type IV includes an appendix found with an inflamed diverticulum [1]. Each subtype is differentiated based on final pathology assessment [1].

Inflammation may be more common with diverticulosis due to the structural change of the appendix [2]. When not inflamed, appendiceal diverticula become difficult to diagnose, as these lesions rarely cause symptoms [4]. When inflamed, however, appendiceal diverticula progress more rapidly to perforation than those with clinical appendicitis without diverticula, therefore appropriate examination is essential [3]. Appendiceal diverticula are most often found in the distal third of the appendix, along the mesenteric border, and commonly present as multiple diverticula [6].

Several studies have noted association between appendiceal diverticula and epithelial neoplasia of the appendix, placing greater importance on post appendectomy specimen pathology for patients who are found to have incidental appendiceal diverticula [1, 3, 4]. Findings of diverticula within an appendectomy specimen should therefore prompt further microscopic and histologic evaluation, to rule out appendiceal neoplasm [5, 6].

## CONCLUSION

Appendiceal diverticula present as rare clinical findings and may often present similarly to acute appendicitis [4, 5]. The diagnosis is often made intraoperatively or post operatively through histopathological evaluation [3]. Preoperative diagnosis is rarely confirmed, but such modalities as radiographic evaluation after barium enema can be greatly helpful [4]. Surgical intervention is both therapeutic and diagnostic [5]. It is crucial to further explore appendectomy specimens, as appendiceal diverticula have been associated with neoplasms [5]. Thus, intraoperative or postoperative management can extend the usual surgical treatment of appendectomy to more complex treatments such as consideration for hemicolectomy [4].
